# The perspective of fluid flow behavior of respiratory droplets and aerosols through the facemasks in context of SARS-CoV-2

**DOI:** 10.1063/5.0029767

**Published:** 2020-11-01

**Authors:** Sanjay Kumar, Heow Pueh Lee

**Affiliations:** Department of Mechanical Engineering, National University of Singapore, 9 Engineering Drive 1, Singapore 117575, Singapore

## Abstract

In the unfortunate event of the current ongoing pandemic COVID-19, where vaccination development is still in the trial phase, several preventive control measures such as social distancing, hand-hygiene, and personal protective equipment have been recommended by health professionals and organizations. Among them, the safe wearing of facemasks has played a vital role in reducing the likelihood and severity of infectious respiratory disease transmission. The reported research in facemasks has covered many of their material types, fabrication techniques, mechanism characterization, and application aspects. However, in more recent times, the focus has shifted toward the theoretical investigations of fluid flow mechanisms involved in the virus-laden particles’ prevention by using facemasks. This exciting research domain aims to address the complex fluid transport that led to designing a facemask with a better performance. This Review discusses the recent updates on fluid flow dynamics through the facemasks. Key design aspects such as thermal comfort and flow resistance are discussed. Furthermore, the recent progress in the investigations on the efficacy of facemasks for the prevention of COVID-19 spread and the impact of wearing facemasks is presented.

## I. INTRODUCTION

The person-to-person transmission of infectious respiratory diseases occurs primarily due to the transportation of virus-laden fluid particles from the infected person. The contagious fluid particles originate from the respiratory tract of the person and are expelled from the nose and the mouth during breathing, talking, singing, sneezing, and coughing.^1–3^ These particles have been broadly classified into two types: aerosols (aerodynamic particle size <5 *μ*m) and droplets (aerodynamic particle size ≥5 *μ*m–10 *μ*m).^4–6^ The finding indicated that the transmission phenomena of these virus particles expelled by patients would be dependent on droplet sizes. Once expelled from the mouth or nose, larger respiratory droplets undergo gravitational settling before evaporation; in contrast, the smaller droplet particles evaporate faster than they settle, subsequently forming the aerosolized droplet nuclei that can be suspended for prolonged periods and travel in the air over long distances. The research studies have revealed that the severe acute respiratory syndrome (SARS) epidemic in 2003 and the current global pandemic of coronavirus disease 2019 (COVID-19) transmitted by contact or through the airborne route.^7–10^ Several preventive strategies such as safe distancing, contact tracing, isolation of the infected person, hand hygiene, and facemasks have been widely employed against the rapid spread of these diseases.^11–14^ Among them, the use of facemasks has proven to be one of the most effective protective measures against airborne virus transmission.^15–20^ The research suggested that face coverings could essentially reduce the forward distance traveled by a virus-laden droplet and thus has great potential to provide personal protection against airborne infection.^21,22^ Recently, the World Health Organization (WHO) has recommended using facemasks for the initial control of COVID-19 spread.^23^

In general, facemasks fall in the category of respiratory protection equipment (RPE) whose primary function is to protect the wearer from airborne viruses and contaminated fluids. There are various RPE types, ranging from simple homemade reusable cloth-based masks to surgical facemasks and N95 respirators to self-contained breathing apparatus.^18,24–27^ Different types of masks provide different levels of protection to the wearer. Surgical facemasks are loose-fitting, fluid-resistant, single-time use, and disposable, designed to cover the mouth and nose. These masks are fluid resistant and intended for reducing the emission of large respiratory droplets released during coughing and sneezing.^28,29^ However, there is a possibility of leakage around the facemask’s edge during the inhaling and exhaling processes. Such a dynamic leakage allows the direct contact of fluid droplets from the outside air to the wearer and vice versa. Such respiratory masks may also not provide adequate protection against extremely fine aerosolized particles, droplets, and nuclei.^30^

For efficient trapping of droplets, the facemask filters should contain microscopic pores; however, the minute-sized pores prevent air ventilation, which creates an uncomfortable situation for the wearer. Hence, a better trade-off between the pore sizes and the breathability is desirable for suitable facemasks. Some mask types that come with inbuilt respirators, such as a filtering facepiece respirator (FFR), P100 respirator/gas mask, self-contained breathing apparatus, full face respirator, and KN95 respirators, provide better breathability for the users. The name designation “N95” in the N95 respirators refers to the filtration of 0.3 *µ*m sized particles with 95% efficiency.^31^ The filtration mechanism of N95 facemasks operates on three possible principles: diffusion, inertial impaction, and electrostatic attraction. The smaller particles (<1 *µ*m) usually get diffused and stuck on the filter’s fibrous layers, whereas particles of typically ≥1 *µ*m get influenced by the inertia effect, preventing them from flowing across the fibers in the filtration layers and get filtered. N95 masks are designed for single-use because of potential contamination of filter layers, resulting in rapid degradation of their filtration efficiency (FE). However, several innovative techniques have been demonstrated for decontaminating and reusing N95 masks.^32,33^ Some polymers such as polypropylene, polyethylene, polyesters, polyamides, polycarbonates, and polyphenylene oxide are usually utilized for the fabrication of N95 filter layers.^34^ However, some recent N95 respirator masks are fabricated using ionic surfactant coated electrocharged polymers or electrospun nanofibers as intermediate layers.^35–37^ These electret fibers trap particles through electrostatic or electrophoretic effects, which help in better filtration of small-size particle transmission.^38–40^

Because of the ongoing COVID-19 pandemic, a significant demand for facemasks has been reported worldwide while stimulating research about their efficacy for filtering expelled droplets from the infected person’s mouth and nose. In this regard, considerable efforts have been made in the past for the evaluation of facemasks’ performance. The quantitative performance of the facemasks has been typically characterized by evaluating the filtration efficiency (FE) and the total inward leakage (TIL).^41–43^ The filtration efficiency refers to the percentage of blocked particles by the tightly fitted facemasks. The filtration efficiency can be calculated as *FE* = (1 − (*C*_*d*_/*C*_*u*_)) × 100%, where *C*_*u*_, *C*_*d*_ are the particle count in the upstream feed prior to filtration and in the downstream filtrate, respectively. TIL is defined as the percentage of particles entering the mask through both the filter and the leakage between the mask and the face. The total inward leakage is calculated by dividing the particle concentrations on the outside and inside the facemasks. The protection factor of the facemasks can be determined from the following expression: PF = 1/TIL. The higher PF value of the masks performs better in virus transmission control.^16^ Furthermore, the fluid penetration resistance performance of the facemasks has been evaluated as per the ASTM F1862/F1862M-17 standards.^44,45^ However, this test method does not evaluate facemasks’ performance for airborne exposure pathways or in preventing the penetration of aerosolized fluids deposited on the facemask. In recent times, some qualitative analysis has been demonstrated for the rapid design characterization of facemasks.^46^

While these experimental studies are essential for the broad characterization and design evaluation of respiratory facemasks, further theoretical and numerical methods and algorithm-based investigations provide a better insight into the facemask’s fluid flow dynamics and the droplet leakage through the facemask openings. If the facemask is donned for a prolonged period, the filtration efficiency may decrease due to the saturation effects.^21^ It has been usually neglected in experimental studies. To involve these factors, an alternative approach, the computational fluid dynamics (CFD) method, can be invaluable for understanding the fluid–particle flow behavior through the facemasks. The fluid dynamics-based numerical techniques have gained momentum in the field of the facemask research domain. The computational fluid flow models have shown their potentials in an improved prediction of the spreading of respiratory virus-laden droplets and aerosols, sensitive to the ambient environment and crucial to the public health responses.^34^

This Review focuses on the fluid flow aspects of the facemasks and their efficacy in virus transmission control. Following a brief introduction to the respiratory infectious diseases and their control strategies (Sec. I), the respiratory droplet transportation mechanisms in conjunction with the possible governing equations required for estimating the transport phenomena are presented in Sec. II. Then, the droplet transport behavior through the facemasks is described in Sec. III. Key design aspects for the facemasks are explained in Sec. IV. Section V covered the recent progress in investigating the efficacy of facemasks for preventing the virus spread. The impact of using the facemasks is discussed in Sec. VI. The concluding remarks and a brief outlook for future research directions are summarized in Sec. VII.

## II. RESPIRATORY DROPLET TRANSPORT GOVERNING EQUATIONS

During the sneezing or coughing process, the dispersion of saliva droplets or aerosols from the mouth to the ambient and eventually on the floor occurs in several stages. The complete transmission cycle involves complex flow phenomena, ranging from air–mucous interaction, breaking of droplets, turbulent conical jets, droplet evaporation and deposition, flow-induced particle dispersion, and sedimentation.^47^ After exhalation from the mouth or nose, the saliva droplet movement is initially led by the inertia force, followed by the formation of a conical jet (vortical flow) near the mouth. Once the droplets are expelled from the mouth, the inertia force gradually decreases, and other forces such as gravity control the dispersion of larger size droplets, while drag and Brownian forces control the smaller size droplets. After traveling up to a particular distance, these virus-laden droplets settle down on the floor.^48^

Thus, there are two major possible pathways for the respiratory virus transmission: airborne inhalation of smaller droplets, which are suspended in ambient air for a more extended period and carried to a longer distance, and contact (direct or indirect between people and with contaminated surfaces) of large size droplets.^49^ The fluid flow behavior of these droplets has been modeled using two different phases: continuous phase for the small size droplet nuclei and discrete phase for large size droplets.

### A. Continuous phase

The fluid flow is governed by the Navier–Stokes and mass transfer equations, which are as follows:

Continuity:

$$\nabla \cdot \vec{u}=0.$$ (1)

Momentum:

$$\rho \frac{\partial \vec{u}}{\partial t}+\rho \left(\vec{u}\cdot \nabla \right)\vec{u}-\mu \nabla^{2}\vec{u}+\nabla p=0.$$ (2)

Mass-transfer:

$$\frac{\partial c}{\partial t}-\psi \Delta c+\vec{u}\cdot \nabla c=0.$$ (3)

Here, $\rho ,\;t,\;\vec{u},\;p,\;\psi ,\;\mu$ denotes the density (kg m^−3^), time (s), flow velocity (m s^−1^), pressure (Pa), diffusion coefficient, and kinematic viscosity, respectively. The conservation laws can be written in the tensor form as follows:

$$\frac{\partial \rho }{\partial t}+\frac{\partial \left(\rho \vec{u_{j}}\right)}{\partial x_{j}}=0,$$ (4)

$$\frac{\partial \left(\rho \vec{u_{j}}\right)}{\partial t}+\frac{\partial \left(\rho \vec{u_{i}}\vec{u_{j}}\right)}{\partial x_{j}}=-\frac{\partial \left(p\right)}{\partial x_{i}}-\frac{\partial \left(\tau_{ij}\right)}{\partial x_{j}}+\vec{S_{f}}.$$ (5)

Here, $\vec{u_{j}}$ represents the flow velocity (m/s) and $\vec{S_{f}}$ is the source term that represents other forces such as gravity and Lorentz force, which also lead to momentum accumulation. For the Newtonian fluids, there is a linear relationship between shear stress and velocity gradient. Hence, the viscous stress tensor can be defined by

$$\tau_{ij}=-\mu {\dot{\gamma }}_{ij}=-\mu \left[\left(\frac{\partial \left(\vec{u_{i}}\right)}{\partial x_{j}}+\frac{\partial \left(\vec{u_{j}}\right)}{\partial x_{i}}\right)\right]+\frac{2}{3}\delta_{ij}\mu \frac{\partial \left(\vec{u_{k}}\right)}{\partial x_{k}}.$$ (6)

In the overall vector form of the constitutive equation,

$$\tau_{ij}=-\mu \left(\nabla \vec{u}+\nabla {\vec{u}}^{T}-\frac{2}{3}\nabla \vec{u}I\right),$$ (7)

where superscript T denotes the transpose of the second velocity gradient outer product. For a Newtonian fluid with constants μ and ρ, the momentum equation can be rewritten as

$$\rho \frac{\partial \left(\vec{u}\right)}{\partial t}=-\nabla p-\mu \left(\nabla^{2}\vec{u}+\nabla \cdot \left(\nabla {\vec{u}}^{T}\right)-\frac{2}{3}\nabla \cdot \left(\nabla \vec{u}I\right)\right)+\vec{S_{f}}.$$ (8)

Moreover, the exhaled fluid jet may contain cough droplets combined with the environmental wind, generating a complex laminar-to-turbulent flow field. The turbulent kinetic energy (K) of the droplets can be obtained by solving the “one equation eddy-viscosity model” (OEEVM) subgrid-scale (SGS),^48,50^

$$\partial \cdot \left(\bar{\rho }k\right)+\nabla \cdot \left(\bar{\rho }ku\right)=-\tau_{ij}\cdot {\dot{\gamma }}_{ij}+\nabla \cdot \left(\mu_{k}\nabla \text{k}\right)+\bar{\rho }\varepsilon ,$$ (9)

$$\varepsilon =c_{\varepsilon }k^{\frac{3}{2}}/\Delta .$$ (10)

The turbulent viscosity is calculated from

$$\mu_{k}=c_{k}\bar{\rho }\Delta \sqrt{k}.$$ (11)

In addition, the fluctuation velocity component for the laminar-to-turbulent airflow field can be predicted by using the Reynolds-averaged Navier–Stokes (RANS) equations’ model,

$${\dot{u}}_{i}=f_{i}\xi_{i}\sqrt{\frac{2}{3}k},$$ (12)

where *ξ*_*i*_ are the damping factors to reflect the anisotropic magnitude of the fluctuation velocity in the near-wall region. These are the random numbers from the standard normal distribution.

The cough spreading phenomena can be predicted by solving the diffusion equation (3) in conjunction with some source and sink terms. Vuorinen *et al.*^51^ developed diffusion-based Monte–Carlo models to realize a transmission phenomenon via the inhalation of aerosols in the ambient flow field. The source and sink terms have been included in conjunction with Eq. (3). The source term represented the transient location of the infected persons, while the sink term has been used for the ventilation surface. The developed models were capable of predicting the aerosol dispersions at more realistic locations such as generic public place and supermarkets where cough may release from the walking person.

### B. Discrete phase (cough droplets’ transport and size change dynamics)

For the droplets with the high droplet-to-air density ratio, the droplet trajectories have been predicted by solving a series of translation equations (Lagrangian approach) of the discrete phase with the assumptions of stationary droplets and limited thermophoresis. Continuous dispersion of saliva droplets throughout the computational domain has been considered in the computations. In addition, some basic parameters such as velocity, mass, and position of each droplet have been computed at every time step. The translational equation for the saliva micro-droplet trajectory is given by^48^

$$\begin{array}{ccc} m_{p}\frac{\partial {\vec{u}}_{P}}{\partial t} & = & {\vec{F}}_{D}+{\vec{F}}_{g}+{\vec{F}}_{L}+{\vec{F}}_{M}\\ & = & \frac{3}{4}C_{D}\frac{\rho_{f}}{\rho_{d}}\frac{m_{d}}{2R_{d}}\left|{\vec{u}}_{f}-{\vec{u}}_{d}\right|\left({\vec{u}}_{f}-{\vec{u}}_{d}\right)+\left(\rho_{d}-\rho_{f}\right)V_{d}\vec{g}+V_{d}\nabla P\\ & & +\;\frac{\rho_{f}V_{d}}{2}\frac{\partial \left({\vec{u}}_{f}-{\vec{u}}_{d}\right)}{\partial t}, \end{array}$$ (13)

where ${\vec{F}}_{D},\;{\vec{F}}_{g},\;{\vec{F}}_{L},\;{\vec{F}}_{BM}$ are the Stokes drag force, gravity, lift or buoyancy force, and Brownian motion-induced force, respectively. In addition, $m_{d},\;R_{d},\;V_{d},\;\rho_{d},\;{\vec{u}}_{d}$ are the mass, radius, volume, density, and velocity vector of the saliva droplets, respectively. $\rho_{f},\;{\vec{u}}_{f}$ are the fluid density and the fluid velocity vector, respectively. The drag coefficient values depend on the droplet’s Reynolds number *Re*_*d*_ and can be calculated from

$$C_{D}=\left\{\begin{array}{cc} 24/Re_{d} & \text{if }Re_{d}<1\\ \left(24/Re_{d}\right)\left(1+0.5R{e_{d}}^{0.687}\right) & \text{if }1\leq Re_{d}\leq 1000\\ 0.44 & \text{if }Re_{d}>1000. \end{array}\right.$$ (14)

Here, $Re_{d}=\frac{2R_{d}\left|{\vec{u}}_{f}-{\vec{u}}_{d}\right|\rho_{f}}{\mu_{f}}$. In the above expressions, the droplet distribution is an important factor as their size decides the travel path distance and eventually the infection risk.^52^ Hence, for the coughing simulation, the droplet breakup approach is used as a sub-model. Pendar and Páscoa^48^ used the Rosin–Rammler breakup approach in their coughing simulation work. In this method, a person’s mouth is modeled by seeding different ranges of droplet radii by invoking a presumed probability density function (PDF) *f*_*r*_, which is expressed as

$$f_{r}=\frac{qr^{q-1}}{{\bar{r}}^{q}}\text{ exp}\left[-{\left(\frac{r}{\bar{r}}\right)}^{q}\right],$$ (15)

where *q*, *r*, and $\bar{r}$ are the exponential factors, drop radius, and average radius of the droplet, respectively. These parameters are based on the saliva injection flow rate as an input for the considered seeding droplet. The above fitting model is also known as Weibull distribution,^53^ which works well for the size distribution of cloud droplets, including water and water-like droplets.^54^

Recently, several studies have attempted to understand the dynamics of droplet formation and transport. Cummins *et al.*^55^ investigated the dispersion of spherical droplets in the presence of a source–sink pair flow field. The Maxey–Riley equation was used to describe the finite-sized spherical particle motion in an ambient fluid flow. The presented non-dimensional mathematical models were based on Newton’s second law of motion in which the forces acting on the particle involved the gravity force, the drag force, an added mass force, the force due to the undisturbed flow, and a Basset–Boussinesq history term. The analytical results suggested that droplets with a smaller size (<75 *µ*m) moved a greater distance because of gravity’s smaller impact. In comparison, the larger size droplets (>400 *µ*m) traveled a relatively long distance before getting pulled into the sink by their more considerable inertia. However, the dispersion of intermediate size droplets (75 *µ*m–400 *µ*m) was found to be complicated under the influence of both the drag and gravity forces. Busco *et al.*^56^ used the computational fluid dynamics approach to predict droplets’ and aerosols’ spread. The biomechanics of a human sneeze, including complex muscle contractions and relaxations, were included in the simulation by imposing a momentum source term to the coupled Eulerian–Lagrangian momentum equations (13). The instantaneous magnitude of the sneezing momentum source term has been defined as |*s*(*t*)| = *p*(*t*)/*L*, where p(t) is the experimental pressure signal and L is the characteristic equivalent length of the human upper-respiratory system ducts. The experimental results validated the developed model for the estimation of droplets and aerosols spreads.

Das *et al.*^57^ investigated the airborne virus transmission through sneezed and coughed droplets and aerosols. The ejected droplet motions were estimated for both still and flowing air conditions by solving the Langevin differential equation using the Monte Carlo numerical method. The Langevin equations for the transport of the droplets of mass (M) in the still air are given as

$$\frac{dr_{i}}{dt}=v_{i},$$ (16)

$$M\frac{dv_{i}}{dt}=-\lambda v_{i}+\xi \left(t\right)+F_{g},$$ (17)

where *dr*_*i*_ and *dv*_*i*_ are the coordinate and velocity shift in each discrete time step *dt*, respectively, and *i* stands for the Cartesian components of the position and velocity vectors. The first term in the right-hand side of Eq. (17) represents the dissipative force. The second term stands for the diffusive (stochastic) force, where ξ(t) is regulated by the diffusion coefficient D. *F*_*g*_ is the gravitation force term acting on a droplet of mass M. In the expression, the value of the drag coefficient *λ* is obtained using the Stokes formula, *λ* = 6*πηR*, where *R* is the droplet radius and *η* is the viscosity. The diffusion coefficient D is obtained from the Einstein relation, *D* = *K*_*B*_*Tλ*, where *K*_*B*_ = 1.38 × 10^−23^ J/K is the Boltzmann constant and T is the temperature in kelvin. As shown, the Langevin differential equations contain a stochastic source term (diffusive force), which is usually ignored in the Eulerian–Lagrangian approach. In addition, environmental factors such as temperature, humidity, and airflow rate, which could influence the air droplet dynamics, were included. The results revealed that the small droplets travel a larger distance and remain suspended in the air for a longer time under the influence of airflow, supporting the mandatory use of facemasks to prevent the virus.

Vadivukkarasan *et al.*^58^ experimentally investigated the breakup morphology of expelled respiratory liquid. The expelled respiratory liquid sputum from a human was emulated using a soap film, and air and flow dynamics were visualized. It was revealed that the droplet formation from the ejected fluid during coughing or sneezing occurred due to three possible mechanisms: Kelvin–Helmholtz (K–H) instability, Rayleigh–Taylor (R–T) instability, and Plateau–Rayleigh (P–R) instability in sequence. The flapping of the expelled liquid sheet was the result of the K–H mechanism, and the ligaments formed on the edge of the rim appeared due to the R–T mechanism, and finally, the hanging droplet fragmentation was the result of the P–R instability.

### C. Droplet evaporation

Droplet evaporation is one of the crucial factors that affects transmission phenomena. The evaporation rate of the droplets depends on the difference between the saturated vapor pressure of the fluid droplet surface and the vapor pressure of the surrounding air (ambient temperature and humidity).^59^ The other factors, such as the mass-diffusion coefficient and the relative velocity between the droplet and the surrounding gas, influence the evaporation rate. The non-dimensional parameters such as Reynolds, Nusselt, and Sherwood numbers govern the droplet evaporation phenomena.^60^ Moreover, the condensation and evaporation effects between the ambient water vapors and the water liquid in cough droplets can be considered by solving the mass and energy balance for each droplet as follows:^61^

Mass balance:

$$\frac{dm_{d}}{dt}=-\overset{k}{\underset{e=1}{\sum }}\int_{surf}n_{e}dA\approx \overset{k}{\underset{e=1}{\sum }}\left({\bar{n}}_{e}dA\right).$$ (18)

Energy balance:

$$\overset{m}{\underset{i=1}{\sum }}m_{d,i}c_{d,i}\cdot \Delta T=\pi d_{d}\lambda_{g}Nu\left(T_{a}-T_{d}\right)-\overset{k}{\underset{e=1}{\sum }}\underset{d}{\iint }n_{e}L_{e}dA.$$ (19)

Here, *n*_*e*_ is the average mass flux of evaporable component *e* on the surface that can be expressed as

$$n_{e}=\frac{\rho_{g}Sh{\tilde{D}}_{e}C_{m}}{d_{d}}\text{ ln}\frac{1-Y_{e,\infty }}{1-Y_{e,surf}},$$ (20)

where *ρ*_*g*_ is the density of the ambient air and *Y*_*e*,*surf*_ and *Y*_*e*,∞_ are the mass fractions of evaporable component *e* on the droplet surface and in the gas phase far from the droplets, respectively. The Sherwood number is calculated as

$$Sh=\sqrt{1+Re_{d}\cdot Sc}\cdot \text{max}\left[1,Re_{d}^{0.077}\right],$$ (21)

where *Sc* = *μ*/*ρD*_*e*_ is the Schmidt number and *D*_*e*_ is the mass diffusivity of component *e*. The Nusselt number is calculated as

$$Nu={\left(1+Re_{d}\cdot Pr\right)}^{0.33}\text{max}\left[1,Re_{d}^{0.077}\right].$$ (22)

Here, *Pr* is the Prandtl number. A detailed explanation of other variables has been given in the previous published article by Feng *et al.*^62^

Several other researchers have studied the flow behavior of evaporating droplets. Recently, Weiss *et al.*^63^ investigated the clustering and evaporation of droplets using the gas phase and droplet coupling equations. The evaporation of droplets and spreading of vapors into the ambient condition were mostly governed by few parameters: the Reynolds number, which is related to the shear rate, the Stokes number, and the mass loading, which is the ratio of the mass of the liquid to the gas phase.^64^ The results suggested that the clustering and evaporation of droplets are primarily affected by the mass loading and Stokes number, while the Taylor-scale Reynolds number was small. When the mass loadings decreased and the Stokes number increased, the droplets dispersed more evenly with a faster evaporation rate. Chaudhuri *et al.*^65^ presented a chemical reaction mechanism-based collision rate model for the prediction of the growth rate of the infected population for the early phases of a COVID-19 like pandemic. Besides, they developed a theoretical model for the aerodynamics of respiratory droplets by considering the evaporation characteristics of levitated droplets. The evolution of the droplets was characterized by a complex interaction of aerodynamics, evaporation thermodynamics, and crystallization kinetics. The fidelity of proposed model was further confirmed by experimentation.

## III. RESPIRATORY DROPLET TRANSMISSION THROUGH THE FACEMASKS

**FIG. 1.**
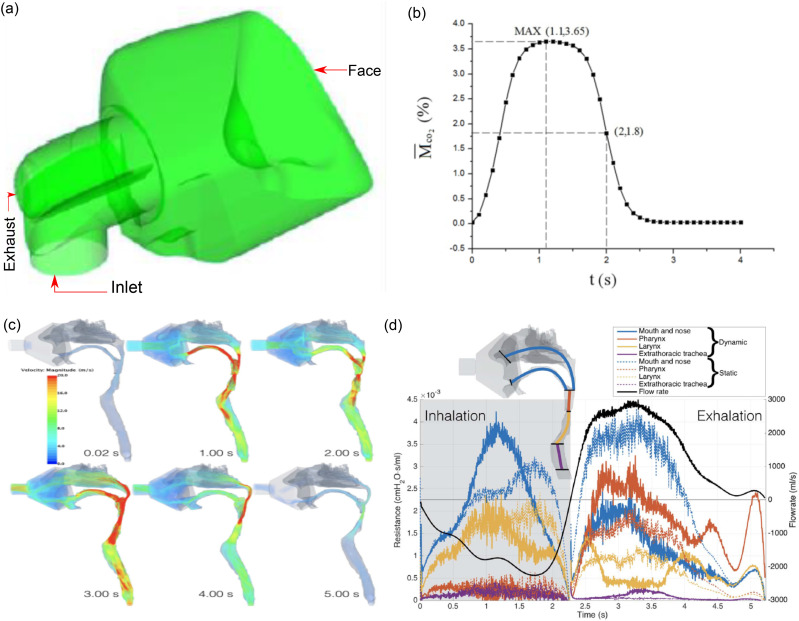
(a) The 3D schematics of the ventilator mask integrated with the volunteer’s face. (b) Distribution of the averaged residual CO_2_ concentration inside the ventilator mask varying with time during a complete respiratory cycle. Reproduced with permission from Zhang *et al.*, “Individualized design of the ventilator mask based on the residual concentration of CO_2_,” Comput. Model. Eng. Sci. **117**, 157 (2018). Copyright 2018 Author(s) licensed under a Creative Commons Attribution 4.0 License. (c) The surface of the airway model at six instants through the breathing maneuver. The model was extended from the mask worn by the wearer. (d) The resistance to airflow through the breath. The colored lines represent the resistance (left axis) through each of the regions between the planes shown in the inset (top left). The solid lines show the resistance in the moving wall simulation, while the dashed lines show the resistance in the same regions in the static geometry. The black curve shows the flow rate throughout the breath (right axis). Reproduced with permission from Bates *et al.*, “Assessing the relationship between movement and airflow in the upper airway using computational fluid dynamics with motion determined from magnetic resonance imaging,” Clin. Biomech. **66**, 88 (2019). Copyright 2019 Elsevier Ltd.

Respiratory droplet transmission is considered critical for the rapid spread and continued circulation of viruses in humans. In recent years, the respiratory droplets’ flow behavior through the facemasks has been typically well-predicted using the computational fluid dynamics (CFD) techniques.^21^ The Navier–Stokes equations have been used as basic governing equations to solve the velocity field in a multi-dimensional computational domain. These equations have been used for the analytical assessment of the respiratory performance of the facemasks and other respirators. Dbouk and Drikakis^21^ performed the fluid dynamics analysis of the respiratory droplets transmission through and around a facemask filter. The compressible Reynolds-averaged Navier–Stokes equations and the k–ω turbulence model were employed. Zhang *et al.*^66^ numerically investigated the carbon dioxide CO_2_ transportation performance inside the ventilator mask. The 3D model of the ventilator mask is shown in Fig. 1(a). The classical Navier–Stokes theorem and mass-transport equations were used to estimate the CO_2_ residual concentrations below the nostrils. The governing equations were solved using the finite volume solver ANSYS fluent 15.0 software. The following boundary conditions were used in the simulation: (i) At the entrance of the ventilator mask, the inlet pressure is 0.98 × 10^3^ Pa and the average concentration of CO_2_ is 0.03%. (ii) At the exhaust holes, outlet pressure is 0 Pa. (iii) The inlet boundary conditions at the nostrils are averaged velocity $\bar{u}=6\times \text{sin}\left(\frac{\pi }{2}t\right)$, expiratory phase time t = 0 s–2.0 s, inspiratory phase time t = 2.0 s–4.0 s, and the averaged concentration of CO_2_ excreted from the nostrils was set as 4%. The airflow inside the ventilator mask was considered to be turbulent flow. Figure 1(b) shows the distribution of the average residual CO_2_ concentration inside the ventilator mask varying with time during a complete respiratory cycle. As shown from the curve, initially, the CO_2_ concentration increased with the increasing exhaled air and reached the peak value of 3.65%, and then, it declined gradually with the decrease in the exhaled air and reached down to the value of 1.8% at the end time of the expiratory cycle. Based on these results, the ventilator mask was redesigned by changing the exhaust hole to the bottom side, and the local residual CO_2_ concentration was decreased to 0.7%.

Bates *et al.*^67^ performed computational fluid dynamics simulations to access the respiratory airflow in the human upper oral airway with airway wall movement. The breathing flow rate data were acquired by imaging the breathing cycle of the participant while wearing a size-5 anesthesia facemask [Fig. 1(c)]. The air pressure drop and flow velocity were estimated by solving the Navier–Stokes equations. In addition, the moving mesh method was used for solving the governing equations. The following interpolation field was used to move all floating mesh vertices in the computational domain:

$$d^{\prime }\left(\vec{x}\right)=\sum_{j=1}^{N}f_{j}\left(r\right)\vec{\lambda_{j}}+\vec{\alpha },$$ (23)

where *d*′ is the calculated displacement applied at each node defined by the position vector $\vec{x}$, N is the number of control points, $\vec{\lambda_{j}}$ is the expansion coefficient, $\vec{\alpha }$ is the constant vector, and *f*_*j*_(*r*) is a radial basis function of the form $f_{j}\left(r\right)=\sqrt{{r_{ij}}^{2}+{c_{j}}^{2}}$. Here, $r_{ij}=\left|\vec{x_{i}}-\vec{x_{j}}\right|$ represented the magnitude of distance between two vertices and *c*_*j*_ the basis constant.

The governing equations (Navier–Stokes equations) in the integral form are given by the following:

Continuity equation:

$$\frac{\partial }{\partial t}\int_{V}\rho \;d\tilde{V}+\oint \rho \left(\vec{u}-\vec{u_{g}}\right)\cdot d\vec{a}=0.$$ (24)

Momentum equation:

$$\frac{\partial }{\partial t}\int_{V}\rho d\tilde{V}+\oint \left(\rho \left(\vec{u}-\vec{u_{g}}\right)⊗\vec{u}\right)\cdot d\vec{a}=-\oint pI\cdot d\vec{a}+\oint T\cdot d\vec{a}.\;$$ (25)

Here, *t* is the time, *V* is the volume of each cell in the mesh, *ρ* is the air density, $\vec{u}$ is the air flow rate, $\vec{u_{g}}$ is the mesh velocity as calculated from the mesh displacement [using Eq. (23)] for each control points, $\vec{a}$ is a vector representing the surface of each mesh cell, ***I*** is the identity matrix, and ***T*** is the viscous stress tensor. These equations were solved using the large eddy simulation (LES) techniques. The instantaneous air flow resistance was calculated as the pressure loss between two locations divided by the air flow rate through them. Figure 1(d) shows the estimated airflow resistance through several different regions of the extra thoracic airway during the complete breathing cycle.

**FIG. 2.**
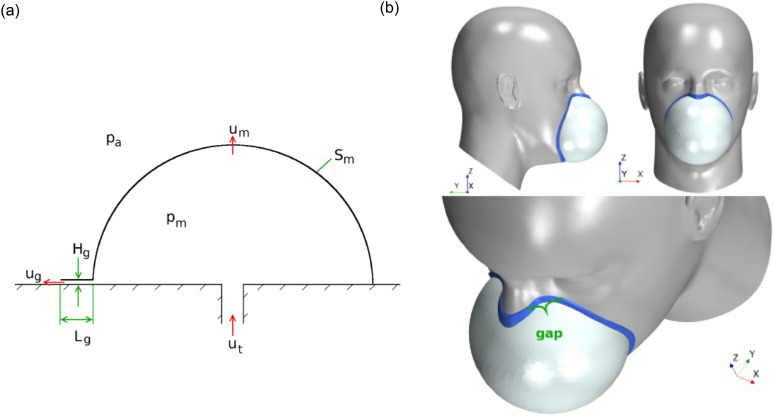
(a) Schematic geometry for airflow through a generic hemi-spherical facemask with gap height *H*_*g*_ and gap length *L*_*g*_ over a width *B*_*g*_ along its perimeter. *u*_*t*_, *u*_*m*_, and *u*_*g*_ denote average airflow velocities through nose cross-sectional area *S*_*t*_, mask filter surface *S*_*m*_, and gap cross-sectional area *S*_*g*_. *p*_*m*_ and *p*_*a*_ signify the pressure inside and outside (atmospheric) of the mask. (b) 3D representation of facemasks with wearer showing the possible region for leakage. Reproduced with permission from R. Perić and M. Perić, arXiv:2005.08800 (2020). Copyright 2020 arXiv.org.

The aerosol–droplet transmission phenomena through the facemasks have also been investigated analytically. The facemask leakage factor has been considered in the analytical models. Lei *et al.*^68^ predicted the fluid leakage between an N95 filtering facepiece respirator (FFR) and a headform using the computational fluid dynamics (CFD) simulation approach. The mass flow rate at the faceseal and through the filter medium was calculated under three different boundary conditions: varying breathing velocity, varying viscous resistance coefficients of the filter, and the freestream air flows. The filter-to-faceseal leakage (FTFL) ratio for the respirator was obtained by dividing the mass flow rate through the filter medium and the faceseal leakage. A higher FTFL ratio refers to the higher percentage of airflow passing through the filter medium than the faceseal leakage. The results revealed the nonlinear increase in the FTFL ratio with an increase in breathing velocity values and a decrease in the filter viscous resistance coefficient values. Furthermore, the freestream flow had limited the influence on the airflow inside the respirator, resulting in nonsignificant variations in the FTFL ratio. Perić *et al.*^69^ investigated the one-dimensional fluid dynamics of the facemasks using analytical and numerical computations. For simplifying the problem, a hemi-spherical geometry was selected for the analysis. Figure 2(a) shows the schematic representation of hemi-spherical facemasks with possible fluid flow directions. When inhaling or exhaling, the total (volumetric) flow rate of the fluid through the nose *F*_*t*_ is given as per mass conservation laws,

$$F_{t}=F_{g}+F_{m}.$$ (26)

The volumetric flow rate can be calculated as *F*_*i*_ = *u*_*i*_*S*_*i*_, where *u*_*i*_ is the average flow velocity and *S*_*i*_ is the cross-sectional area. In the expression, the subscript *i* denotes the nose (*t*), airgap (*g*), and mask filter (*m*). Moreover, the fluid flow through the gap is considered as fully developed laminar Poiseuille flow because the average gap velocity is estimated to be below the critical velocity $u_{crit}\approx \frac{\upsilon Re_{crit}}{D_{h}}$, where *D*_*h*_ is the hydraulic diameter and *Re*_*crit*_ is the Reynolds number. In addition, fluid passes through the gap between the face and the facemasks, and a pressure drop can be observed at the inlet, inside the gap, and at the outlet. The pressure drop at the gap inlet and outlet is given as

$$\Delta P_{g,1}=\frac{u_{t}}{\left|u_{t}\right|}\xi \frac{\rho }{2}u_{g}^{2}.$$ (27)

The pressure drop inside the gap is given by

$$\Delta P_{g,2}=\frac{12\mu L_{g}}{H_{g}^{2}}u_{g}.$$ (28)

The total pressure drop through the gap must be equal to the pressure drop through the mask filter-piece,

$$\Delta P_{m}=\Delta P_{g,1}+\Delta P_{g,2},$$ (29)

where Δ*P*_*m*_=*C*_*m*_*ρ*_*f*_*u*_*m*_, *C*_*m*_ is the viscous porous resistance of the mask filter material, *ρ*_*f*_ is the fluid density, and *u*_*m*_ is the average flow velocity through the mask that can be computed from the expression *F*_*m*_ = *u*_*m*_*S*_*m*_. Equations (26)–(29) are used for the theoretical estimation of fluid flow behavior.

## IV. KEY DESIGN ASPECTS

### A. Thermal comfort

**FIG. 3.**
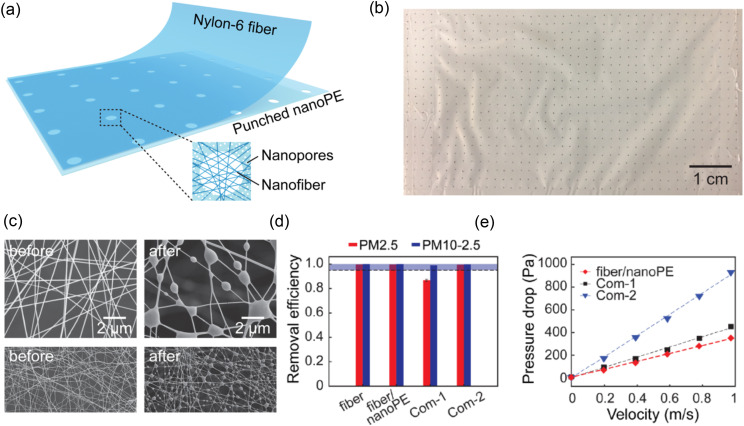
[(a) and (b)] Schematics of the proposed hybrid facemask (nanofibers/nanoPE) and its photograph. (c) The SEM images show the condition of nylon-6 fibers before and after filtering the particulate matter (PM). [(d) and (e)] The removal efficiency of the fiber/nanoPE facemasks compared to two commercial masks, and their pressure drop spectra as a function of the wind velocity. Reproduced with permission from Yang *et al.*, “Thermal management in nanofiber-based face mask,” Nano Lett. **17**, 3506 (2017). Copyright 2017 American Chemical Society.

Thermal comfort is an essential aspect of a facemask as it may affect the compliance of the use of the facemask during summer or in tropical countries. There were reported incidence of skin rashes, increased heat stress, sweating, and discomfort due to prolonged wearing of a facemask in hot and humid conditions.^70^ To improve the thermal comfort level of facemasks, researchers have developed some unique facemasks by using the nanocomposites. Polymer-based nanofibers with a large surface area-to-volume ratio have shown great potential for use in facemasks to achieve both high filtration efficiency and sufficient air permeability.^71–73^ Yang *et al.*^74^ presented a design of the nanofiber-based facemasks for a better thermal comfort of the user. The facemask was made of hybrid nanocomposites containing electrospun nylon-6 nanofibers on top of the needle-punched nanoporous polyethylene (nanoPE) substrate. While nanofibers with strong particulate matter (PM) adhesion properties ensured high PM capture efficiency (99.6% for PM2.5) with a low pressure drop, a nanoPE substrate with high infrared (IR) transparency (92.1%, weighted based on human body radiation) resulted in effective radiative cooling. Figures 3(a)–3(c) show the schematic, photographs, and scanning electron micrographs of the proposed hybrid nanofiber-based facemask. The comparative PM capture efficiency and air permeability results have demonstrated the superiority of the presented facemask over the commercial masks [Figs. 3(d) and 3(e)]. Moreover, the thermal image revealed that the fiber/nanoPE facemasks had high transparency to the human body radiation (cooling effect). In contrast, the commercial facemasks blocked a large portion of it. They further modified the nanoPE substrate with Ag coating and demonstrated that fiber/Ag/nanoPE had a warming effect.

Zhang *et al.*^75^ reported the use of an active ventilation fan to reduce the dead space temperature and CO_2_ level. An infrared camera (IRC) method was used to elucidate the temperature distribution on the prototype FFR’s outside surface and the wearer’s face, and surface temperature was found to be lowered notably. Both the inside and outside temperatures resulted from the simulation were found to be in good agreement with experimental results. However, the inward blowing fans may compromise the filtering effectiveness of the facemask. There are commercially available facemasks fitted with a one-way valve for facilitating the removal of humidity and expired air within the space between the facemask and the face. However, during the COVID-19 pandemic, one of the main reasons for wearing the mask is not only to protect the inhalation of the virus but also to prevent the spread of the virus into the air if the wearer happens to be a carrier of the virus. If the wearer is a healthy subject, the use of a one-way valve and ventilation fan would, indeed, mitigate the buildup of humidity and carbon dioxide within the dead space. Zhu *et al.*^76^ reported a three-dimensional model of a normal human nasal cavity to simulate the volume of fraction (VOF) of both fresh air and respired air within the nasal cavity. The model consisted of a large rectangular domain outside the nasal cavity representing ambient air, human nasal cavity, and partial of the pharynx. This was the first reported piece of work that modeled the details of nasal cavity instead of just the nostrils as openings for the flow simulations. The advantage of this simulation was that the flow field within the space between the nostrils and the facemask could be more accurately simulated as the boundary condition could be specified away from the nostril at the pharyngeal area. Two cases were simulated. Case I refers to a human face with a N95 respirator onto the human face, and case II refers to a human face without a respirator. The results showed that above 60% of inspired air was respired air in case I compared to less than 1.2% in case II. During expiration, the volume of fraction (VOF) of respired air in both the cases was above 95%. The streamlines at peak inspiration were relatively smooth while entering the cavity in both the cases; while at peak expiration, large vortex was observed within the air space between the human face and the respirator in case I. For future studies, one could explore the *in vivo* experimental studies with the use of miniaturized and wireless sensors for monitoring not just the temperature but also the humidity and carbon dioxide content within the space between the nostrils and the facemask. The sensors need to be small so as not to disrupt the flow fields. If a single sensor cannot be small enough for the measurement of all the three parameters, one may need to have separate sensors and repeat the experiment for the same human subject.

### B. Flow resistance

Another important parameter affecting the comfort of the wears is the flow resistance of the facemask. In principle, if the flow resistance is lower while maintaining the same filtering efficiency, the comfort level will be enhanced. However, the facemask’s flow resistance is just an indicator and does not specify the wearer’s breathing resistance. While the flow resistance could be measured using a typical setup for correlating the fluid flow rate to the pressure drop across the facemasks, the breathing resistance could only be measured using a human subject or a replica of the nasal pharyngeal system. Lee and Wang^77^ presented the pioneering work of measuring the nasal airflow resistance during inspiration and expiration using a standard rhinomanometry and nasal spirometry. A modified full-facemask was produced in-house to measure nasal resistance using N95 (3M 8210) respirators. The results showed a mean increment of 126% and 122% in inspiratory and expiratory flow resistances, respectively, with N95 respirators. There was also an average reduction of 37% in air exchange volume with the use of N95 respirators.

The same group did a follow-up study investigating the change in human nasal functions after wearing an N95 respirator and a surgical facemask.^78^ The human subject study involved 87 healthy healthcare workers. Each of the volunteers attended two sessions and wore an N95 respirator in session 1 (S1) and surgical facemask in session 2 (S2) for 3 h. The mean minimum cross-sectional area (mMCA) of the two nasal airways via acoustic rhinometry and nasal resistance via rhinomanometry was measured before and immediately after the mask. The equipment could not be used to perform *in vivo* measurement with the facemask on. Rhinomanometry was repeated every 30 min for 1.5 h after the removal of masks. A questionnaire was distributed to each of the volunteers during the 3 h mask-wearing period to report subjective feelings on the discomfort level of breathing activity. Among 77 volunteers who completed both the two sessions, the mean nasal resistance immediately increased upon removing the surgical facemask and N95 respirator. The mean nasal resistance was significantly higher in S1 than S2 at 0.5 h and 1.5 h after removing the masks (p < 0.01). There was an increase in nasal resistance upon the removal of the N95 respirator and surgical facemask potentially due to nasal physiological changes. The N95 respirator caused higher post-wearing nasal resistance than the surgical facemask with different recovering routines. This was the first time that the effect of long duration wearing of a facemask was objectively monitored. However, the duration of 3 h for wearing a facemask was deemed to be too short under the current COVID-19 simulations, and a human subject study for a longer duration of wearing a facemask should be attempted. The research could also be enhanced using miniaturized pressure, temperature, humidity, and gas sensors for *in vivo* monitoring of the air condition within the space between the nostrils and the facemask. Such experimental data would be useful for validating numerical models for assessing the comfort level for wearing different types of facemask. Another potential approach is to develop a replica for replacing a human subject for such a long duration study, similar to the use of an acoustic head for replacing human subjects in the more extended duration noise exposure study.

Zhu *et al.*^79^ reported another investigation on the effect of long duration wearing of N95 and surgical facemasks on upper airway functions. A total of 47 volunteers of National University Hospital of Singapore participated for the study. Each of the volunteers wore both the N95 respirator and the surgical facemask for 3 h on two different days. During the period of mask wearing, relative airflow rates were recorded. The study revealed the increased level of discomfort to the user with time while wearing the masks. Moreover, the N95 respirator caused higher post-wearing nasal resistance than the surgical facemask with different recovering routines.

## V. EFFECTIVENESS OF FACEMASKS FOR PREVENTION OF VIRUS TRANSMISSION

The current studies recognized the airborne transmission of aerosols produced by asymptomatic individuals during speaking and breathing as a key factor, leading to the spread of infectious respiratory diseases such as COVID-19.^62,80–82^ However, the spread of these airborne diseases has been successfully controlled up to a certain extent by using the facemasks.^11,19,49,83–85^ In the ongoing global pandemic of the COVID-19, where vaccine development is still at a phase of the trial stage, the respiratory protective equipment such as facemasks has proven to be a complementary countermeasure against the spread of the novel coronavirus. In this regard, several researchers have performed theoretical and experimental investigations of virus transmissibility through the facemasks and alternatives. Stutt *et al.*^86^ developed the holistic mathematical frameworks for assessing the potential impact of facemasks in COVID-19 pandemic management. The results revealed that professional and home-made facemasks were highly efficacious to reduce exposure to respiratory infections among the public. In addition, when people wear the facemasks all-time at the public places, the certain epidemiological threshold, known as the effective reproduction number, could be decreased below 1, leading to the prevention of epidemic spread. Ngonghala *et al.*^87^ developed a parametric model for providing deeper insights into the transmission dynamics and control of COVID-19 in a community. They used the COVID-19 data from New York state and the entire US to assess the population-level impact of various intervention strategies. The results suggested that the consistent use of facemasks could significantly reduce the effective reproduction number. The highly efficacious facemask, such as surgical masks with an estimated efficacy of around 70%, could lead to the eradication of the pandemic if at least 70% of the residents use such masks in public consistently. The use of low efficacy masks, such as cloth masks with an estimated efficacy of 30%, could also lead to a significant reduction of COVID-19 burden. Yan *et al.*^88^ evaluated the effectiveness of different respiratory protective equipment in controlling infection rates in an influenza outbreak. They used a previously developed risk assessment model^89^ to show N95 respirators’ efficacy, low-filtration surgical mask (adult), high-filtration surgical mask (adult), high filtration pediatric mask, and low filtration pediatric mask. The study revealed that donning these masks with a 50% compliance rate resulted in a significant reduction in transmission risk and with 80% compliance rate nearly eradicated the influenza outbreak. Prasanna Simha and Mohan Rao^90^ quantitatively investigated the distance of travel of typical human coughs with and without different masks: disposable three-ply surgical masks and N95 masks. In their study, the schlieren method, a highly sensitive, non-intrusive flow imagining technique, was used to visualize the human cough flow features. The experimental statistics showed that the propagation of a viscous vortex ring mainly governed cough flow behavior. While wearing regular face masks, the cough droplets traveled approximately half the distance traveled by expelled droplets without a mask. However, N95 was found to be most effective in limiting the spread of cough droplets. Leung *et al.*^91^ performed experimental studies to investigate the efficacy of surgical facemasks to prevent respiratory virus shedding. The surgical facemasks’ efficiency was measured against the coronavirus, influenza virus, and rhinovirus of two broad particle sizes, respiratory droplets (≥5 *μ*m) and aerosols (droplet nuclei with aerodynamic diameter ≤5 *μ*m). The results indicated that surgical facemasks could efficaciously prevent the transmission of human coronaviruses and influenza viruses into the environment in respiratory droplets, but no significant reduction in aerosols.

**FIG. 4.**
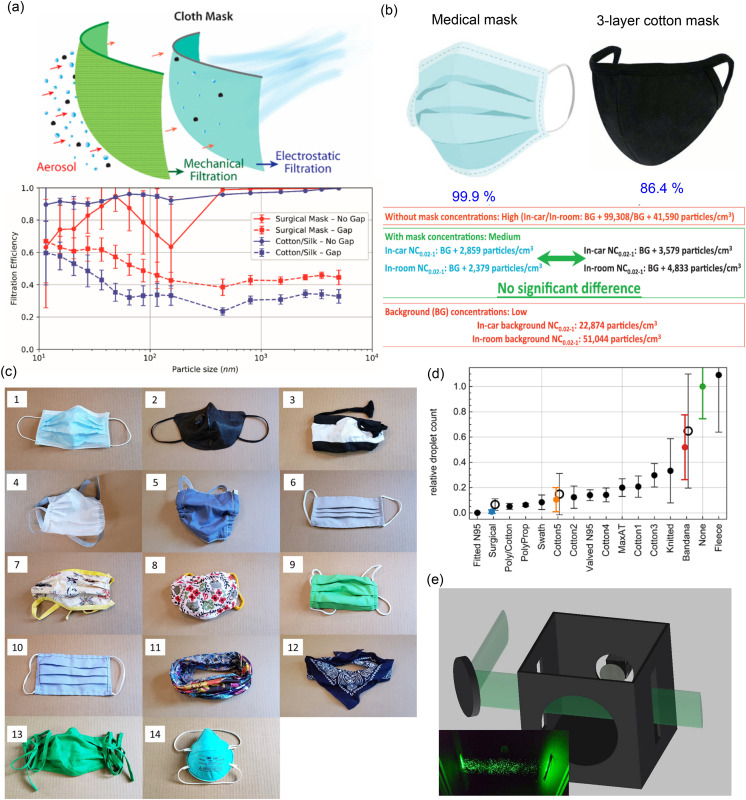
(a) Schematic illustration of the possible filtration mechanism of the hybrid cloth masks. In addition, the plot shows the filtration efficiencies of a surgical mask and hybrid fabric cotton/silk with (dashed) and without a gap (solid). The gap used was ∼1% of the active mask surface area. Reprinted with permission from Konda *et al.*, “Aerosol filtration efficiency of common fabrics used in respiratory cloth masks,” ACS Nano **14**, 6339 (2020). Copyright 2020 American Chemical Society. (b) Performance comparison between the medical masks and the three-layer cotton mask. Reproduced with permission from Ho *et al.*, “Medical mask versus cotton mask for preventing respiratory droplet transmission in micro environments,” Sci. Total Environ. **735**, 139510 (2020). Copyright 2020 Elsevier B.V. (c) Photographs of the facemasks under investigation: (1) three-layer surgical mask, (2) N95 mask with a exhalation valve “Valved N95,” (3) knitted mask, (4) double-layer polypropylene apron mask “Polyprop,” (5) cotton–polypropylene–cotton mask “Poly/cotton,” (6) single layer Maxima AT mask “MaxAT,” (7) double-layer cotton-pleated style mask “Cotton2,” (8) double-layer cotton mask–Olson style mask “Cotton4,” (9) double-layer cotton-pleated style mask “Cotton3,” (10) single-layer cotton-pleated style mask “Cotton1,” (11) gaiter type neck fleece “Fleece,” (12) double-layer bandana “Bandana,” (13) single-layer cotton-pleated style mask “Cotton5,” and (14) N95 mask no exhalation valve fitted “Fitted N95.” (d) Relative droplet transmission through the corresponding facemasks. (e) Schematic of the experimental optical setup. Reproduced with permission from Fischer *et al.*, “Low-cost measurement of face mask efficacy for filtering expelled droplets during speech,” Sci. Adv. **6**, eabd3083 (2020). Copyright 2015 Author(s), licensed under a Creative Commons Attribution 4.0 License.

Moreover, the steep rise in demand for medical facemasks during the current pandemic COVID-19 has resulted in a subsequent breakdown of the global supply chain that led to an acute shortage in the market. To mitigate this discontinuous supply chain system, scientists have put much effort into exploring alternative fabrics with sufficient filtering capacity that are readily available and affordable. Kähler and Hain^92^ performed a detailed analysis of the efficacy of facemasks to prevent virus spread. In the first step, the transmission of droplets released by the mouth when breathing, speaking, and coughing was characterized. Then, the filtering capacity of the various facemasks was analyzed. The experimental results have shown that most household materials tested do not provide much protection against the virus transmission via droplets and, therefore, are unsuitable as materials for protective masks. However, filtering facepiece respirator (FFR) performance-based masks such as FFP2 (Europe EN 149-2001), N95 (United States NIOSH-42CFR84), DS2 (Japan JMHLW-Notification 214, 2018), and KN95 (China GB2626-2006) offer adequate protection as they are only permeable to a tiny fraction of few micrometer-sized droplets. Konda *et al.*^93^ evaluated the filtration efficiency of various commonly available fabrics, including cotton, silk, chiffon, flannel, various synthetics, and their combinations, which were used in the fabrication of cloth masks. The filtration performance of these fabrics was conducted by generating the aerosol particles at the cloth sample’s upstream side. The aerosol particulates ranging from ∼10 nm to ∼10 *μ*m scale sizes, particularly relevant for respiratory virus transmission, were produced by using a commercial sodium chloride (NaCl) aerosol generator. In addition, the air with a controlled airflow rate was drawn through the sample using a blower fan. The filtration efficiency *η*_*f*_ of each sample was computed by measuring the particles’ concentration upstream and downstream as $\eta_{f}=\frac{C_{u}-C_{d}}{C_{u}}\times 100$, where *C*_*u*_ and *C*_*d*_ are the mean particle concentrations per bin upstream and downstream, respectively. Moreover, the pressure drop across the facemasks and the air velocities were measured using a digital manometer and a hot wire anemometer. The experimental investigations revealed that the materials such as natural silk, a chiffon weave (90% polyester–10% Spandex fabric), and flannel (65% cotton–35% polyester blend) provided good electrostatic filtering of particles. In addition, fabric with tighter weaves and low porosity, such as cotton sheets with a high thread count, has resulted in better filtration efficiencies. For instance, a 600 TPI (thread per inch) cotton sheet can provide average filtration efficiencies of 79 ± 23% (in the 10 nm–300 nm range) and 98.4 ± 0.2% (in the 300 nm to 6 *µ*m range). A cotton quilt with batting provides 96% ± 2% (10 nm–300 nm) and 96.1 ± 0.3% (300 nm to 6 *µ*m). Surprisingly, a four-layer silk (e.g., scarf) was found to be effective with an average filtration efficiency of >85% across the 10 nm to 6 *µ*m particle size range. Moreover, the hybrid masks made by combinations of two or more fabric types, leveraging mechanical and electrostatic filtering, could be an effective approach for better filtration [Fig. 4(a)]. Verma *et al.*^46^ performed the qualitative investigations for assessing the effectiveness of easily available facemasks such as bandana (elastic T-shirt material, 85 threads/in.), folded handkerchief (cotton, 55 threads/in.), stitched mask (quilting cotton, 70 threads/in.), and other commercial masks. They observed that a stitched mask made of quilting cotton was most effective, followed by the commercial mask, the folded handkerchief, and, finally, the bandana. Their observations also suggested that a higher thread count by itself is not sufficient to provide a better droplet filtration capability. The material types and fabrication techniques have a significant impact on the performance of facemasks. Davies *et al.*^42^ examined the efficacy of homemade masks as an alternative to commercial surgical masks. Various household materials such as 100% cotton T-shirt, scarf, tea towel, pillowcase, antimicrobial pillowcase, vacuum cleaner bag, cotton mix, linen, and silk were evaluated for the capacity to prevent bacterial and viral aerosol transmission. The performance of these household facemasks was compared with the standard surgical mask. The experimental outcomes showed that these homemade masks could reduce the likelihood of infection but are not efficient for the complete elimination of risks. A similar conclusion has been made in a previously published review article by Rossettie *et al.*^94^ and Loupa *et al.*^95^ Recently, Ho *et al.*^96^ investigated the droplet filtration efficiency of the self-designed triple-layer cotton masks, and their performance was compared with the standard medical mask. All tests were performed in two different locations: in a regular bedroom and a car with air conditioning. The particles with a size range of 20 nm–1000 nm were taken into consideration, and the filtration efficiency was measured. Other factors such as environmental conditions (temperature and relative humidity) and cough/sneeze counts per hour were measured for each measurement. The results revealed that cotton and surgical masks could significantly reduce the number of microorganisms expelled by participants with the filtration efficiency of 86.4% and 99.9%, respectively [Fig. 4(b)]. However, the surgical mask was three times more effective in blocking transmission than the cotton mask. In a recent study, Fischer *et al.*^97^ performed testing of 14 different facemasks or mask alternatives ranging from the kind worn by healthcare professionals to neck fleeces and knitted masks. Figure 4(c) shows the photographs of the facemasks and alternatives considered in the investigation. A comparison was made on the dispersal of droplets from a mask wearer’s breath while wearing one of the face coverings to the results of a controlled trial where their mouth was fully exposed. The study revealed that some mask types matched standard surgical masks’ performance, while some mask alternatives, such as neck fleece or bandanas, offered little protection against infection [Fig. 4(d)]. Besides, they demonstrated a simple optical measurement method to evaluate the efficacy of facemasks to reduce respiratory droplet transmission during regular speech. Figure 4(e) shows the schematic of the developed setup. The proposed optical system is inexpensive and easy-to-operate, even by non-experts.

Furthermore, the use of face shields has widely been used along with standard face masks. Face shields are generally made of transparent plastic sheets. They offer several advantages as follows: comfortable to wear, easy-to-clean, clear conversations between the speakers with visible facial expressions, and reduce autoinoculation by preventing the wearer from touching their face.^98^In addition, face shields prevent the user’s face from the direct contact of liquid droplets. More recently, Verma *et al.*^99^ investigated the effectiveness of the face shields and exhalation valves in the respiratory droplet transport context. They performed experimentation in an emulated coughing and sneezing environment for a qualitative visualization analysis. The results indicated that although face shields block the initial forward motion of the fluid jet, the expelled droplets can move around the visor with relative ease and spread out over a large area depending on environmental conditions. In addition, for the facemasks equipped with an exhalation port, the droplets pass through the exhalation valves. Based on the observations, they opined that high-quality cloth or surgical masks perform better than the face shields and exhalation valves.

## VI. IMPACT OF USING FACEMASKS AND RECENT DESIGNS

In the past few decades, especially post-outbreak of the severe acute respiratory syndrome (SARS) in 2003, wearing the facemasks has grown extensively. The people from Asian countries, such as China, Singapore, Thailand, and Japan, can be easily seen donning facemasks in public places. There are well-proven studies about the prevention of airborne pathogen transmission by covering the mouth and nose using the facemasks. The recently published article by Gandhi and Rutherford^100^ claimed that the universal facial masking might help reduce the severity of disease and enhance the wearer’s immunity. In addition, there has been an apprehension about the carbon dioxide build up during the prolonged wearing of a facemask; however, the recent experimental studies contradicted this myth. According to the reported clinical observation study by Samannan *et al.*,^101^ the wearing of facemasks neither significantly restricted the gas exchange (oxygen circulation) nor contributed to carbon dioxide buildup, even in persons with severe lung impairment.

Nevertheless, prolonged use of facemasks has some side effects on human respiratory health, such as thermal stress, drowsiness, breathing problems because of restricted fresh airflow, and unusual heart rate.^102,103^ The discomfort felt with surgical mask use has also been ascribed to neurological reactions or associated psychological phenomena such as anxiety, claustrophobia, or affective responses to the perceived difficulty in breathing.^101^ In addition, if a facemask is donned for a longer period, the filter gets wet because of facial sweat, and vapor is formed inside the facemasks due to the breathing, resulting in clogging of particulates. In addition, wearers get a false sense of security, encouraging them to spend more time in public places.^104^ Other potential side effects of facemasks’ wearing include skin irritation, uncomfortable feeling due to the arrival of exhaled air into the eye, comprised quality, and the volume of the speech during the conversations.^19,105,106^

Moreover, there are some environmental concerns associated with the use of single-use facemasks. Some of these facemasks are made from plastics layers, which may not bio-degrade easily, thus creating a massive burden on the environment. A recent analysis has reported that if every person in the UK used one single-use facemask each day for a year, it would create 66 000 tonnes of contaminated plastic waste, roughly 10 times higher than using reusable masks.

The new coronavirus is continuously evolving and spread all over the world. Researchers from all disciplines, especially medical professionals and engineers, are continuously working on facemask design improvement for a better performance against the virus transmission. Cheng *et al.*^107^ presented an electrospun polyetherimide (PEI) electret nonwoven material-based bi-functional smart facemask to remove sub-micrometer particulate matter and generate electricity. The facemask could harvest sufficient energy from the airflow to supply power to the inbuilt LCD panel. The LCD screen was used to display the measured breathing rate. Hossain *et al.*^40^ developed a rechargeable N95 facemask composed of a charged polypropylene electret fiber made up of an intermediate layer for capturing the foreign particles. These particles are trapped through the electrostatic or electrophoretic effects of the polypropylene terephthalate (PET) layer. The mask has a provision for the *in situ* recharging of the polypropylene electret for maintaining its filtration performance. Williams *et al.*^108^ proposed a facemask used for sample collection of the respiratory SARS-CoV-2 virus. They have successfully presented a facemask prototype that detects exhaled *Mycobacterium tuberculosis*, a deadly lung infection, and are now working on sampling of the SARS-CoV-2 virus. The facemask consisted of four 3D printed polyvinyl alcohol (PVA) sampling strips attached inside it. The sampling matrices trapped the particulates during exhalation and were further post-processed for the virus diagnosis. Face-mask sampling offered a highly efficient and non-invasive method for a respiratory disease diagnosis. The presented approach showed great potential for diagnosis and screening, particularly in resource-limited settings.

Moreover, several innovative facemask prototypes with better filtration performance are available on the market. Recently, Korean electronics and appliance company LG® Ltd. has developed an air purifier wearable mask (PuriCare^TM^)^109^ equipped with battery-operated miniature fans that draw in the fresh air and help reduce stuffiness. The researchers of the Massachusetts Institute of Technology and Brigham and Women Hospital, Boston have developed a silicone-based transparent reusable facemask with a comparable performance level with N95 respirators.^110^

## VII. SUMMARY

The facemasks have shown their potential for preventing the spread of respiratory diseases. A variety of facemasks ranging from a simple homemade cloth mask to the ventilated respirators have played their role in the current COVID-19 pandemic. In general, the facemasks have been experimentally characterized by determining the filtration efficiency and total inward leakage ratio. In addition, the fluid flow dynamics-based numerical methods have gained much attention for investigating the facemask performances. The present article has also highlighted the insufficiencies in assessing the breathing resistance of the wearers with the facemask by just examining the flow resistance of the facemask. In the long term, there may be a need for a more elaborate system approach, including the study and modeling of how the human lung would respond to the increase in breathing resistance due to the use of a facemask, drawing the analogy of modeling the behavior of the heart for the blood circulation system. This article summarizes the perspective of the fluid dynamics of the facemask filtration performance, including droplet and aerosol transports, droplet evaporation, and facemask aerodynamics. Furthermore, recent investigations for the efficacy of the facemasks in the context of respiratory virus transmission have been discussed.

## DATA AVAILABILITY

The data that support the findings of this study are available from the corresponding author upon reasonable request.
